# Supplementary material for: The Delayed Turnover of Proteasome Processing of Myocilin upon Dexamethasone Stimulation Introduces the Profiling of Trabecular Meshwork Cells’ Ubiquitylome
Source: Int J Mol Sci. 2024 Sep 17;25(18):10017. doi: 10.3390/ijms251810017 (PMC11432723; doi:10.3390/ijms251810017)
Supplement: Supplementary file 1 [file ijms-25-10017-s001.zip › ijms-3163446-supplementary.pdf]

Figure 1

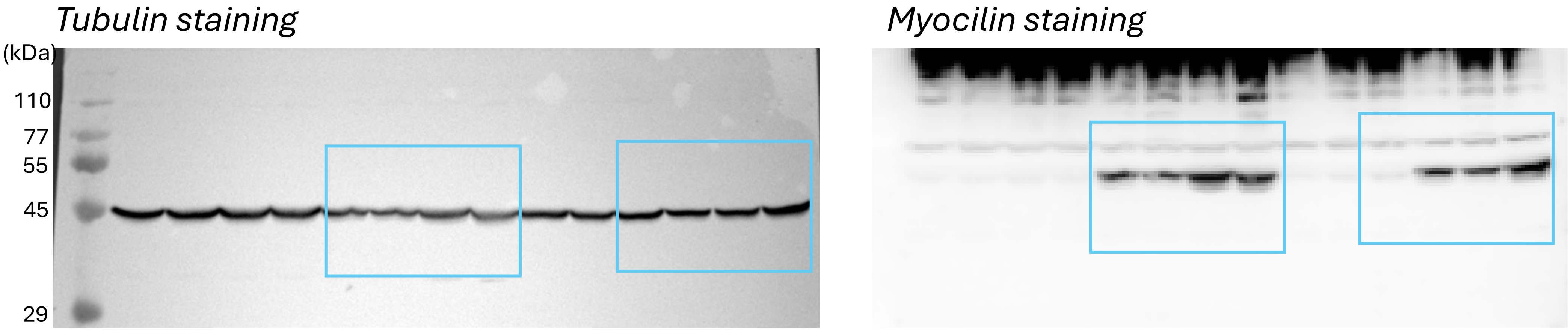

Figure 2 Myocilin and Ub Staining – Native Gel

Myociln Low exposure

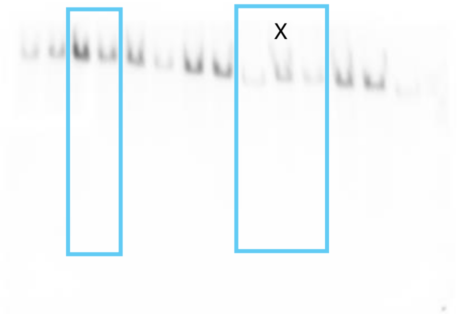

X: identifies a cropped lane

Myocilin High exposure

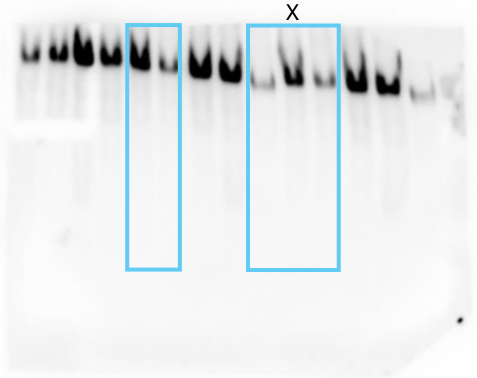

X: identifies a cropped lane

Ubiquitin staining

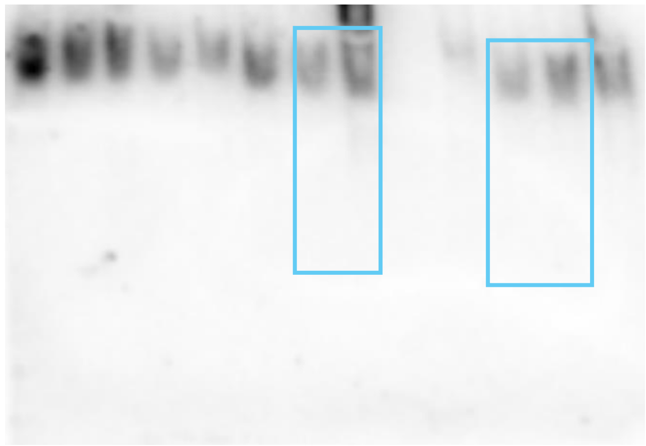

Figure 4

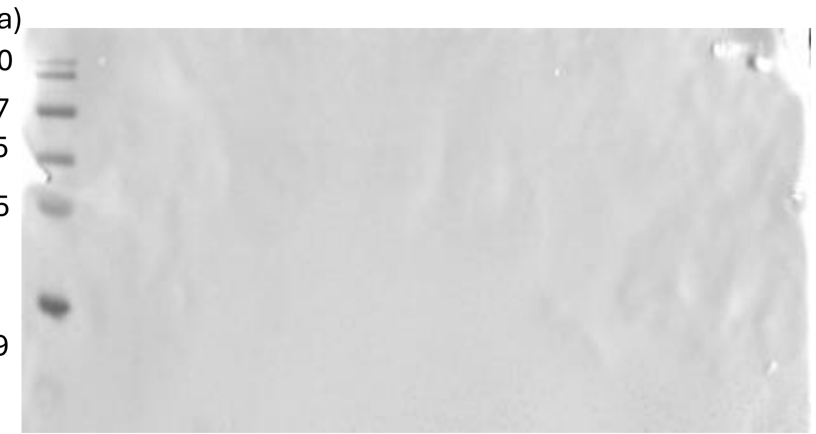

CHIP/STUB1 staining

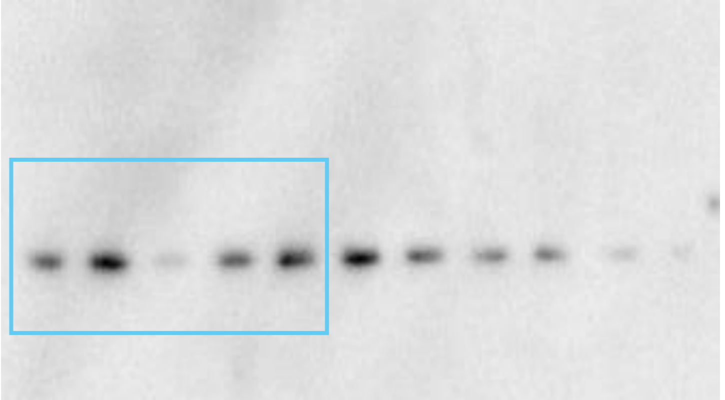

Calnexin staining

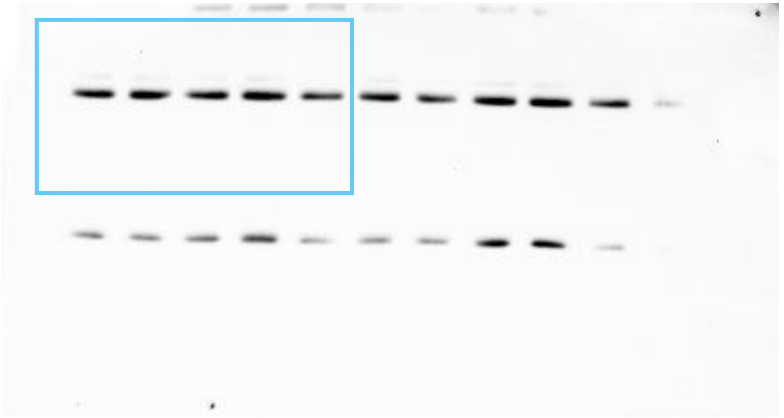

GAPDH staining

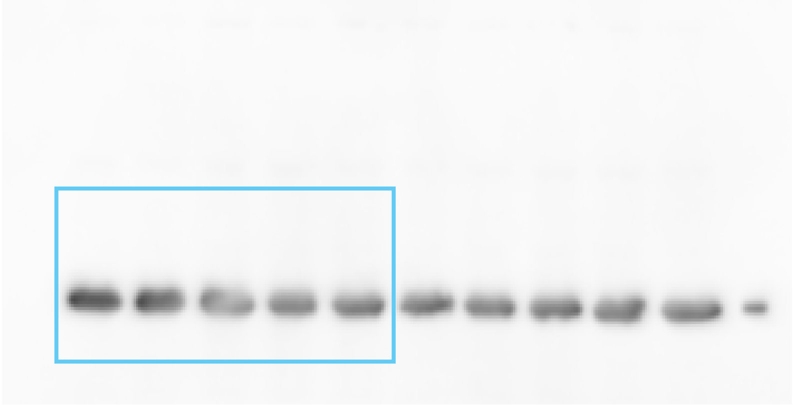

Myocilin staining

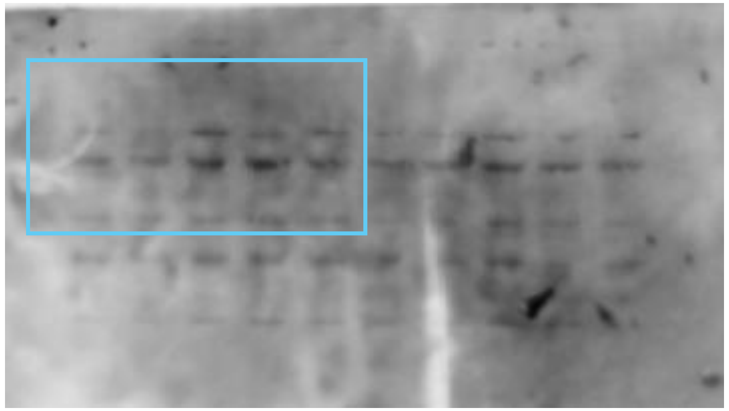

S Ponceau Staining - Supernatant

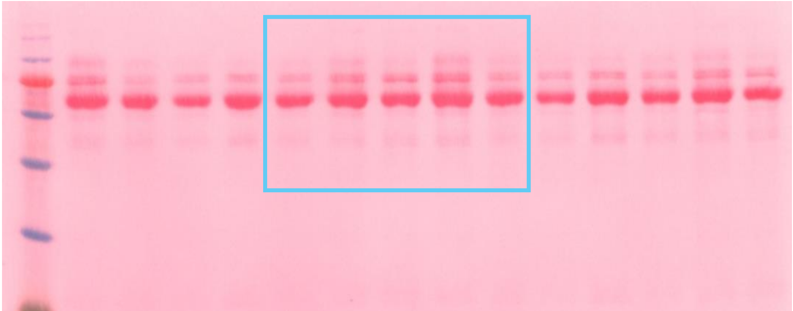

Myocilin staining - Supernatant

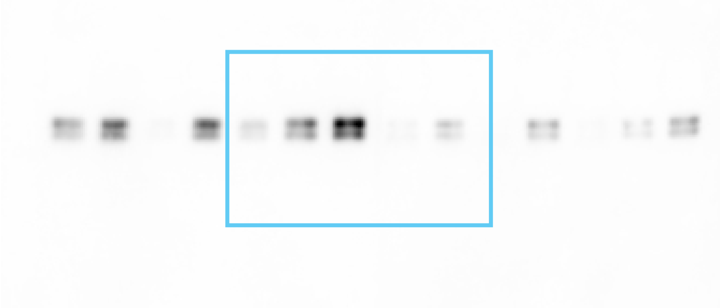

Figure 5

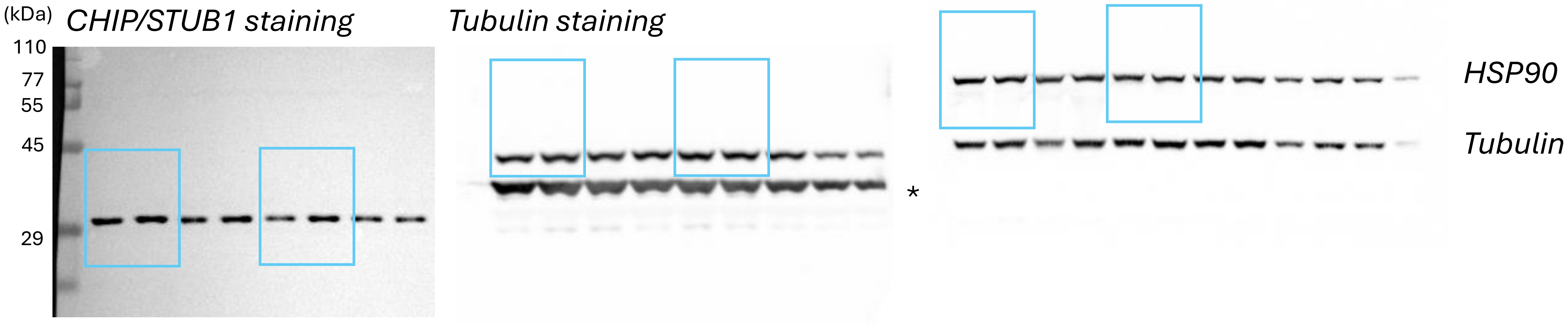

* The band indicated by the asterisk corresponds to beta-actin which was first probed highlighting that saturation was reached
